# Extent of late gadolinium enhancement detected by cardiovascular magnetic resonance correlates with the inducibility of ventricular tachyarrhythmia in hypertrophic cardiomyopathy

**DOI:** 10.1186/1532-429X-12-30

**Published:** 2010-05-21

**Authors:** Stephan Fluechter, Jürgen Kuschyk, Christian Wolpert, Christina Doesch, Christian Veltmann, Dariusch Haghi, Stefan O Schoenberg, Tim Sueselbeck, Tjeerd Germans, Florian Streitner, Martin Borggrefe, Theano Papavassiliu

**Affiliations:** 1 1st Medical Department, Medical Faculty Mannheim, Heidelberg University, Mannheim, Germany; 2 Institute of Clinical Radiology and Nuclear Medicine, Medical Faculty Mannheim, Heidelberg University, Mannheim, Germany; 3 VU University Medical Center, Amsterdam, the Netherlands

## Abstract

**Background:**

Myocardial fibrosis is frequently identified in patients with hypertrophic cardiomyopathy (HCM). The aim of this study was to investigate the role of myocardial fibrosis detected by late gadolinium-enhancement (LGE) cardiovascular magnetic resonance (CMR) as a potential arrhythmogenic substrate in HCM. We hypothesized that the extent of LGE might be associated with the inducibility of ventricular tachyarrhythmias (VT) during programmed ventricular stimulation (PVS).

**Methods:**

We evaluated retrospectively LGE CMR of 76 consecutive HCM patients, of which 43 presented with one or more risk factors for sudden cardiac death (SCD) and were therefore clinically classified as high-risk patients. Of these 43 patients, 38 additionally underwent an electrophysiological testing (EP). CMR indices and the extent of LGE, given as the % of LV mass with LGE were correlated with the presence of risk factors for SCD and the results of EP.

**Results:**

High-risk patients had a significant higher prevalence of LGE than low-risk patients (29/43 [67%] versus 14/33 [47%]; p = 0.03). Also the % of LV mass with LGE was significantly higher in high-risk patients than in low-risk patients (14% versus 3%, p = 0.001, respectively). Of the 38 high- risk patients, 12 had inducible VT during EP. LV function, volumes and mass were comparable in patients with and without inducible VT. However, the % of LV mass with LGE was significantly higher in patients with inducible VT compared to those without (22% versus 10%, p = 0.03). The prevalence of LGE was, however, comparable between HCM patients with and those without inducible VT (10/12 [83%] versus 15/26 [58%]; p = 0.12). In the univariate analysis the % of LV mass with LGE and the septal wall thickness were significantly associated with the high-risk group (p = 0.001 and 0.004, respectively). Multivariate analysis demonstrated that the extent of LGE was the only independent predictor of the risk group (p = 0.03).

**Conclusions:**

The extent of LGE in HCM patients correlated with risk factors of SCD and the likelihood of inducible VT. Furthermore, LGE extent was the only independent predictor of the risk group. This supports the hypothesis that the extent of fibrosis may serve as potential arrhythmogenic substrate for the occurrence of VT, especially in patients with clinical risk factors for SCD.

## Background

Sudden cardiac death (SCD) can be the first and most devastating clinical manifestation of hypertrophic cardiomyopathy (HCM). Ventricular tachyarrhythmias (VTs) seem to be the principal mechanisms of SCD in HCM patients [1]. Thus, identifying individuals at high risk for SCD is of paramount importance. Risk stratification of patients with HCM is mainly based on clinical markers, such as an unexplained syncope [2], non sustained VT [3,4], a family history of HCM and SCD [5], and presence of severe left ventricular hypertrophy [6].

Although scarred myocardium is an established anatomic and electrophysiological substrate for the occurrence of VT and SCD in patients with coronary artery disease [7,8], its role in HCM is less clear. Evidence for slowed and fragmented intraventricular conduction as seen in patients with ischemic heart disease has also been observed in patients with HCM, and these observations have been associated with risk for SCD [9-11]. Slowed conduction in HCM, although attributed to potential electrophysiological effects of the structural disruption and myocardial disarray, may simply reflect the substrate of myocardial scarring as in ischemic heart disease [12]. Late gadolinium-enhanced (LGE) cardiovascular magnetic resonance (CMR) allows visualization of myocardial scarring in HCM [13,14]. Recent studies in patients with HCM proposed LGE as a predisposing factor for disease progression and SCD using clinical risk factors as surrogates for clinical endpoints [15]. Further studies found LGE to be most common in HCM patients with VT indicating that LGE may play some role in increasing arrhythmic risk mediated by nonsustained VT [16-18].

The aim of the study was to investigate the role of LGE as a potential arrhythmogenic substrate in HCM. Thus, we hypothesized that the extent of LGE is enlarged in high risk patients for SCD and might be associated with the inducibility of VTs during programmed ventricular stimulation (PVS). The results of PVS were therefore used as surrogate for characterising a potential arrhythmogenic substrate as visualised by LGE CMR and correlated to the CMR findings.

## Methods

### Study population

A total of 76 patients with HCM (48 males and 26 females; mean age 57 ± 14 years) were evaluated between February 2003 and December 2009 by CMR at the 1^st ^Department of Medicine and the Institute of Clinical Radiology and Nuclear Medicine, University Hospital of Mannheim, Germany. These patients fulfilled conventional criteria for HCM with LV hypertrophy ≥ 15 mm on two-dimensional echocardiography in the absence of another disease that could cause the hypertrophy [19]. The work-up at initial diagnosis included electrocardiogram, echocardiography, coronary angiography, left ventriculography, 24-h Holter ECG and CMR imaging.

Five clinical risk factors for SCD were used to stratify the study population in high- and low-risk patients: history of cardiac arrest, a family history of sudden cardiac death, unexplained syncope or presyncope, documented non-sustained ventricular tachycardia and presence of severe left ventricular hypertrophy ≥ 30 mm [20]. 43 of 76 patients (28 males and 15 females; mean age 56 ± 15 years) presented one or more risk factors for SCD and were therefore classified as high-risk patients. 38 of these 43 patients additionally underwent a complete electrophysiological study including programmed ventricular stimulation (PVS). The remaining 5 patients refused to undergo an electrophysiological study.

Informed consents for the CMR and the electrophysiological testing protocol were obtained from all subjects. The performance of this study was consistent with the standards of the local ethical committee at our institution.

### CMR acquisition

All studies were performed using a 1.5 Tesla whole body imaging system (Magnetom Sonata, Siemens Medical Solutions, Erlangen, Germany). A dedicated four-element, phased-array body coil was used. Images were acquired during repeated end-expiratory breath-holds. Scout images (coronal, sagittal and axial planes) were obtained for planning of the final double-oblique long-axis and short-axis views. To evaluate functional parameters, ECG-gated cine images were then acquired using a segmented steady state free precession (trueFISP) sequence. Typical scan parameters were: 5 mm slice thickness with 5 mm interslice gap, temporal resolution 35 ms, repetition time 3.2 ms, echo time 1.2 ms, flip angle of 60 degrees, and typical in-plane spatial resolution 1.4 × 1.8 mm^2^. A stack of 9 to 12 short-axis slices was used for full coverage of the left and right ventricle.

**Figure 1:**
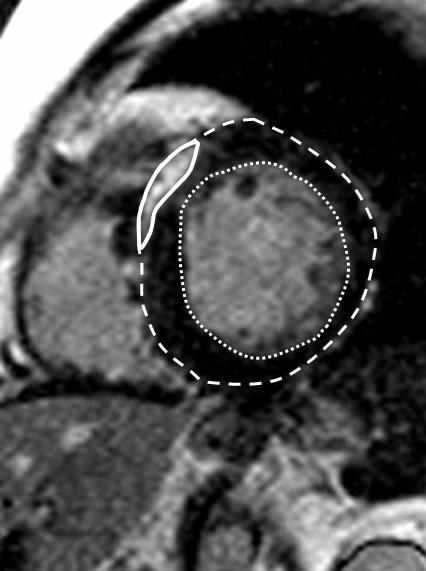
**Late gadolinium enhancement (LGE) in a short axis view of a patient with hypertrophic cardiomyopathy: Endocardial (......) and epicardial (-----) contours**. % of LV mass with LGE assessed by visual planimetry (thick line).

The LGE images were obtained 10 min after intravenous administration of 0.2 mmol/kg gadolinium-DTPA (Magnevist, Schering AG, Berlin, Germany). An inversion recovery turbo Fast Low Angle Shot (FLASH) sequence or a fast multislice single-shot 2D IR true fast imaging with steady-state precession sequence were used to obtain images at the same position as the long- and short-axis cines in end-diastole (Figure 1) [21,22]. The inversion time was adjusted per patient to optimally null signal from normal myocardium (typically 250 to 300 ms). Total acquisition time averaged 40 minutes.

### Image analysis and determination of ventricular and atrial parameters

CMR images were analysed both qualitatively and quantitatively by two experienced investigators blinded to the electrophysiological findings. LV end-diastolic volumes, LV end-systolic volumes, LV stroke volume and LV ejection fraction were assessed off-line from the serial short-axis trueFISP cine loops using dedicated commercially available software (ARGUS, Siemens, Erlangen, Germany). In addition to volumetric measurements, one-dimensional measurements of LV end diastolic dimensions, posterior wall thickness and maximum interventricular septum wall thickness were measured from end diastolic short-axis views.

The LGE was assessed visually, and the LGE mass was measured by manual planimetry on all short-axis slices by 2 observers blinded to all patient details (SF; TP) (Figure 1). Summing the LGE mass of all slices yielded the total mass of LGE. The extent of LGE was then expressed as a percentage of the total LV mass (the % of LV mass with LGE).

### Programmed ventricular stimulation

43 of the 76 patients presented one or more risk factors for SCD. 38 of these 43 high-risk patients underwent a complete electrophysiological study including programmed ventricular stimulation. Antiarrhythmic drugs were discontinued for at least five half-lives before electrophysiological study. Two bipolar or quadripolar catheters were introduced percutaneously through the right or left femoral vein and positioned at the high right atrium and the right ventricular apex under fluoroscopic guidance. PVS was performed using a Biotronik UHS-20 stimulator (Biotronik GmbH & Co. KG). Pulses of 1.9 ms in duration were applied at twice diastolic threshold.

### Electrophysiological study protocol

Assessment of sinus node recovery time, atrial and atrioventricular node refractoriness was performed with a conventional stimulation protocol. PVS was performed with up to three extrastimuli at three different driving cycle lengths (500 ms, 430 ms, and 370 ms) at the right ventricular apex and the right ventricular outflow tract until refractoriness. VT/VF were defined as sustained when the duration exceeded 30 s or required cardioversion for hemodynamic collapse.

### Statistical analysis

All data are presented as a mean ± standard deviation. For comparing left and right ventricular parameters and LGE-extent in different patients groups an unpaired, 2-tailed student's t-test was used. A Chi-square test was used to evaluate if the presence of LGE, risk factors for SCD and clinical symptoms were different between different patients groups.

Multivariate analysis was performed with logistic regression analysis using block entry of the following variables: extent of LGE and septal wall thickness to evaluate if these variables were independent predictors of the risk group in HCM patients, provided to have a p < 0.05 in univariate analysis.

All results were considered statistically significant when p < 0.05. Analyses were performed with Statistical Package for Social Sciences (SPSS for windows 14.0, Chicago, IL, USA).

## Results

There were no statistically significant differences regarding sex and age between patients at high- and low- risk for SCD (Table 1). Table 1 also shows the values for all LV parameters for patients at high- and low-risk for SCD measured by CMR and the clinical characteristics. Besides a significantly thickened septum wall in high risk patients, there were no statistically significant differences in LV-function and volumes between high- and low-risk patients. High-risk patients showed a significantly higher prevalence of LGE than low-risk patients (29/43 [67%] versus 14/33 [47%]; X^2 ^= 4.69, p = 0.03). Additionally, the % of LV mass with LGE was significantly higher in high- than in low-risk patients (14% vs. 3% in low-risk patients; p = 0.01) (Table 1).

**Table 1:** CMR parameters and clinical characteristics of patients at high- and low-risk for sudden cardiac death

	Patients at High risk	Patients at Low-risk	*p-values*
n	43	33	
male gender	28	20	*0.50*
Age	57 ± 14	61 ± 10	*0*.29
***CMR parameters***			
EF (%)	57 ± 4	62 ± 10	*0.09*
EDM (g)	195 ± 58	181 ± 76	*0.36*
EDV (ml)	156 ± 53	139 ± 42	*0.14*
ESV (ml)	67 ± 32	54 ± 28	*0.07*
SV (ml)	88 ± 30	84 ± 28	*0.58*
LVEDD (mm)	50 ± 7	49 ± 7	*0.54*
SWT (mm)	20 ± 5	17 ± 4	**0.004**
PWT (mm)	10 ± 3	10 ± 2	*0.91*
RVEDD (mm)	43 ± 6	42 ± 7	*0.42*
RVESD (mm)	28 ± 6	28 ± 7	*0.76*
Presence of LGE	29/43 (67%)	14/33 (47%)	**0.03**
			
% of LV mass with LGE	14%	3%	**0.001**
***Clinical characteristics***			
Dyspnea	11	4	*0.14*
Chest pain	13	5	*0.12*
Atrial fibrillation	18	3	*0.26*
HCM with obstruction	25	10	*0.06*
Diabetes	4	5	*0.35*
Hypertension	25	19	*0.49*
Hyperlipidemia	10	13	*0.13*

EDM = enddiastolic mass; EDV = enddiastolic volume; EF = ejection fraction; ESV = endsystolic volume; LGE = Late gadolinium enhancement; LV = left ventricular; Presence of LGE = Number of patients in whom LGE was detectable; LVEDD = left ventricular enddiastolic diameter; PWT = posterior-wall-thickness; RVEDD/RVESD = right ventricular enddiastolic/enddiastolic diameter; SV = stroke volume; SWT = Septal-wall-thickness; (Mean value ± standard deviation).

In Table 2 patients at high risk for SCD were further subdivided in patients with and without inducible ventricular tachyarrhythmias during PVS. There were no statistically significant differences in LV-function and volumes between patients with and without inducible tachyarrhythmias. Patients with inducible ventricular tachycardias showed a higher prevalence of LGE than patients without (10/12 = 83% versus 15/26 = 58%), but this difference did not reach statistical significance (chi square test: X^2 ^= 2.34, p = 0.12). The % of LV mass with LGE, however, was significantly higher in patients with inducible ventricular tachyarrhythmias compared to those without (22% versus 10%, p = 0.03).

**Table 2:** CMR parameters of high risk patients with and without inducible VT/VF during EP

	Patients with inducible VT/VF (*)	Patients without inducible VT/VF (*)	*p-values*
**n**	12	26	
**male gender**	7	17	*0.45*
**age**	60 ± 11	54 ± 16	*0.34*
			
**EF (%)**	58 ± 13	59 ± 10	*0.81*
**EDM (g)**	202 ± 58	195 ± 59	*0.32*
**EDV (ml)**	169 ± 60	150 ± 48	*0.32*
**ESV (ml)**	75 ± 45	63 ± 26	*0.31*
**SV (ml)**	94 ± 29	85 ± 30	*0.41*
**LVEDD (mm)**	50 ± 9	51 ± 7	*0.85*
**SWT (mm)**	22 ± 4	19 ± 5	*0.13*
**PWT (mm)**	10 ± 3	11 ± 4	*0.34*
**RVEDD (mm)**	44 ± 5	43 ± 7	*0.63*
**RVSD (mm)**	27 ± 7	28 ± 6	*0.45*
			
**Presence** **of LGE**	10/12 (83%)	15/26 (58%)	*0.12*
			
**% of LV mass with LGE**	22%	10%	**0.03**

^(*) ^38 of 43 High risk patients underwent EP-testing; EDM = enddiastolic mass; EDV = enddiastolic volume; EF = left ventricular ejection fraction; ESV = endsystolic volume; LGE = Late gadolinium enhancement; Extent of LGE(%) = Extent of LGE expressed as a percentage of total myocardium; Presence of LGE = Number of patients in whom LGE was detectable (irrespective of extent); Segments with LGE = Number of segments according to the AHA 17-segments system that show LGE; LVEDD = left ventricular enddiastolic diameter; PWT = posterior-wall-thickness; RVEDD = right ventricular enddiastolic diameter; RVESD = right ventricular endsystolic diameter; SV = stroke volume; SWT = Septal-wall-thickness; (Mean value ± standard deviation).

Clinical characteristics and risk factors of patients at high risk for SCD are presented in table 3. There were no statistical differences concerning the incidence of any of the listed criteria between patients with inducible VT/VF and patients without. However, 7 out of 12 patients with inducible tachyarrhythmias had two or more risk factors for SCD, compared to only 5 in the group of patients without inducible VT/VF (p = 0.04).

**Table 3:** Risk factors for SCD and clinical characteristics of high risk patients with and without inducible ventricular tachyarrhythmias.

	Inducible VT/VF (*)	No-inducible VT/VF (*)	p-values
n	12	26	
**Risk factors for sudden cardiac death**			
Aborted sudden death	1	0	*0.14*
Family history of SCD	2	3	*0.45*
Syncope or presyncope	8	17	*0.49*
Documented nsVT	6	9	*0.31*
Wall thickness > 30 mm	2	1	*0.16*
2 or more risk factors	7	5	**0.04**
			
**Clinical characteristics and symptoms**			
Dyspnea	3	6	*0.49*
Chest pain	5	7	*0.30*
Palpitations	3	6	*0.49*
Atrial fibrillation	6	10	*0.38*
HCM with obstruction	7	15	*0.49*
Diabetes	2	1	0.16
Hypertension	8	15	0.42
Hyperlipidemia	3	5	*0.45*

^(*) ^38 of 43 High risk patients underwent EP-testing; nsVT = non sustained ventricular tachycardia; SCD = sudden cardiac death; VF Ventricular Fibrillation; VT = Ventricular Tachycardia;

In the univariate analysis the % of LV mass with LGE and the septal wall thickness were significantly associated with the high-risk group in HCM patients (p = 0.001 and 0.004, respectively). The multivariate logistic regression analysis, a model using these both parameters to predict the high risk group in HCM patients revealed that only the % of LV mass with LGE was independently associated with the high risk group in HCM patients (p = 0.03).

### PVS results in high-risk patients

Sustained ventricular tachyarrhythmias were inducible in 12 of 38 patients (32%). Polymorphic VT and VF were inducible in 7 and 5 patients, respectively (Table 4). In 26 patients no sustained tachycardia was inducible.

**Table 4:** Electrophysiological characteristics of HCM patients with inducible ventricular tachyarrhythmias

Patient Nr.	age	sex	Mode of induction	Induced arrhythmia	Average cycle length of induced arrhythmia	Site of induction
1	56	w	370/S2S3	polymorphic VT	220	RV Apex
2	72	m	500/S2S3	polymorphic VT	288	RV Apex
3	68	m	500/S2S3S4	polymorphic VT	168	RV Apex
4	65	w	500/S2S3	polymorphic VT	224	RVOT
5	71	w	430/S2S3S4	polymorphic VT	240	RV Apex
6	55	m	500/S2S3S4	polymorphic VT	192	RV Apex
7	76	w	370/S2S3	polymorphic VT	212	RV Apex
8	38	m	500/S2S3S4	VF		RV Apex
9	51	m	500/S2S3S4	VF		RVOT
10	53	m	500/S2S3S4	VF		RVOT
11	57	m	500/S2S3S4	VF		RV Apex
12	59	w	500/S2S3S4	VF		RV Apex

HCM = Hypertrophic cardiomyopathy; RV = Right ventricle; RVOT = Right Ventricular Outflow Tract; VT = Ventricular Tachycardia; VF = Ventricular Fibrillation

## Discussion

The present study demonstrates with state-of-the-art CMR that HCM patients at high risk for SCD show a significantly higher prevalence and % of LV mass with LGE compared to low-risk patients. Moreover high-risk patients with inducible ventricular tachyarrhythmias during PVS displayed significantly more LGE compared to patients without inducible tachyarrhythmias. However, the presence of LGE was not related to the inducibility of ventricular tachyarrhythmias. Additionally, LGE extent was the only independent predictor of the risk group. These findings provide further evidence in support of the hypothesis that myocardial fibrosis is an important arrhythmogenic substrate in patients with HCM.

The histological basis for LGE in patients with HCM is currently believed to be increased collagen deposition [13]. Focal areas of fibrosis caused by myocardial scarring or interstitial fibrosis may serve as anatomical substrate for the occurrence of ventricular tachyarrhythmias. Moon et al found a relation between the extent of LGE and clinical risk factors for SCD in patients with HCM [15]. This observation is in line with our results, where high risk patients presented a significantly higher % of LV mass with LGE than patient without risk factors for SCD.

In our retrospective study the results of PVS were additionally used to further characterise patients at high risk for SCD. In literature the value of electrophysiological testing in HCM is discussed controversially so that current guidelines do not recommend electrophysiological testing in risk stratification of HCM [23]. Nevertheless Fananapazir et al. could show that patients with inducible VT had a poorer prognosis than those without inducible arrhythmias [24].

Our results show a correlation between the inducibility of ventricular tachyarrhythmias and the extent of LGE in patients with HCM. The findings of this study are consistent with recent reports of the correlation between risk of arrhythmia and the presence and morphology of scar in other patients groups such as those with ischemic or non ischemic cardiomyopathy: Schmidt et al. demonstrated in a high-risk population of patients with prior myocardial infarction and LV dysfunction, that tissue heterogeneity detected by LGE-CMR correlates with an enhanced susceptibility to ventricular arrhythmia during EP or device testing [25]. Bello et al. could point out that in patients with known coronary artery disease, infarct surface area and mass defined by LGE-CMR were better identifiers of inducible monomorphic VT during electrophysiological testing than LV ejection fraction alone [26]. Additionally, Nazarian and al. found out, that the distribution of scar identified by CMR is also predictive of inducible VT in patients with nonischemic cardiomyopathy [27].

Teraoka et al. correlated the extent of LGE with the occurrence of ventricular tachyarrhythmias in patients with HCM. The group with VT/VF showed significantly more often LGE (p < 0.05) and the areas of LGE were significantly larger (p = 0.03) [16]. In this context, Varnava et al. examined the pathologic findings in 75 patients with HCM in relation to morphology, clinical features, and patient outcome. Myocardial fibrosis was markedly increased in patients who had documented nonsustained VT or a high risk fractionation study, suggesting that fibrosis rather than disarray is the anatomic substrate for sudden death [28]. Since LGE can non-invasively identify fibrotic tissue in patients with HCM, the findings of Varnava et al. underline the potential prognostic value of CMR imaging in patients with HCM.

Accordingly, Adabag and colleagues recently found that premature ventricular contractions, couplets and nonsustained ventricular tachycardia on 24-h ambulatory Holter electrocardiograms were more common in patients with HCM and LGE than in patients without. Within the group of LGE the risk of ventricular arrhythmias was not correlated to the extent of LGE [17]. In our present analysis, the mere presence of LGE was not related to the inducibility of ventricular tachyarrhythmias. The lack of association between presence of LGE and inducibility of ventricular tachyarrhythmias was unexpected. There was a distinct trend toward a higher prevalence of LGE in patients with inducible ventricular tachyarhythmias, but this difference did not reach statistical significance, since the study seems underpowered for this comparison. Only the extent of LGE correlated significantly with the inducibility of ventricular tachyarrhythmias. One further possible explanation for the differences between the two studies might be owing to the different composition of the cohorts. Our patients were older (mean-age of 57 ± 14 years vs. 41 ± 16 years) and showed a higher prevalence of LGE (57% vs 41%). Additionally, inducibility of ventricular tachyarrhythmias is a different denominator or clinical endpoint than PVC, or NSVT and therefore can not be directly compared.

### Study limitations

We performed a retrospective observational study with a rather small sample size. Additionally, only patients who were clinically classified as high risk patients for SCD were examined by EP. Therefore this study population contains a selection of symptomatic patients which does not reflect the broad spectrum of patients with HCM.

At present there is no general consensus on the strategy how to exactly define the extent of LGE in HCM. In our study we analyzed LGE-extent by visual assessment. According to our own experience and to recent studies by Adabag et al. and Bondarenko et al. visual estimation yields comparable results to assessment with 5- or 6-SD threshold techniques whereas strategies thresholding at 2-SD may result in an overestimation of LGE-extent [17,29].

## Conclusions

HCM patients with clinical risk factors for SCD show a significantly higher prevalence and larger extent of LGE compared to low-risk patients. Moreover the likelihood of inducible ventricular tachyarrhythmias increases with the extent of LGE in HCM patients at high risk for SCD. Additionally, LGE extent was the only independent predictor of the risk group. These findings provide further evidence in support of the hypothesis that the extent of fibrosis potentiates the arrhythmogeneity in HCM and underline a potential predictive value of LGE CMR in patients with HCM as an adjuvant to current risk stratification protocols.

## Abbreviations

HCM: Hypertrophic cardiomyopathy; LGE: Late gadolinium enhancement; CMR: Cardiovascular magnetic resonance; SCD: Sudden cardiac death; EP: Electrophysiological Testing; LV: Left ventricular; VT: Ventricular tachycardia; PVS: Programmed ventricular stimulation; VF: Ventricular fibrillation.

## Competing interests

The authors declare that they have no competing interests.

## Authors' contributions

SF is the corresponding author, performed and assessed CMR images, participated in study-design, statistical analysis, figures and tables and composed the manuscript. JK equally contributed to the composition of the manuscript, evaluated results of electrophysiological testing and wrote the passage concerning electrophysiological testing. CW participated in study-design and performed and evaluated electrophysiological testing. CD Participated in collection and evaluation of clinical data. CV performed and evaluated electrophysiological testing and helped in statistical analysis. DH Participated in collection and evaluation of clinical data. SOS participated in study-design and assessment of CMR-images. TS participated in study-design. TG participated in study-design, manuscript composition and evaluation of CMR data. MB participated in study-design, scientific and clinical advice concerning HCM. FS helped in statistical analysis and collection of clinical data. TP performed and assessed CMR images, participated in study-design and the composition of the manuscript. All authors read and approved the final manuscript.
